# Convolutional Neural Networks for Classifying Laterality of Vestibular Schwannomas on Single MRI Slices—A Feasibility Study

**DOI:** 10.3390/diagnostics11091676

**Published:** 2021-09-14

**Authors:** Philipp Sager, Lukas Näf, Erwin Vu, Tim Fischer, Paul M. Putora, Felix Ehret, Christoph Fürweger, Christina Schröder, Robert Förster, Daniel R. Zwahlen, Alexander Muacevic, Paul Windisch

**Affiliations:** 1 Department of Radiation Oncology, Kantonsspital Winterthur, 8400 Winterthur, Switzerland; psager@outlook.com (P.S.); christina.schroeder@ksw.ch (C.S.); robert.foerster@ksw.ch (R.F.); daniel.zwahlen@ksw.ch (D.R.Z.); 2 Department of Radiology, Kantonsspital St. Gallen, 9007 St. Gallen, Switzerland; Lukas.Naef@kssg.ch (L.N.); Tim.Fischer@kssg.ch (T.F.); 3 Department of Radiation Oncology, Kantonsspital St. Gallen, 9007 St. Gallen, Switzerland; Erwin.Vu@kssg.ch (E.V.); PaulMartin.Putora@kssg.ch (P.M.P.); 4 Department of Radiation Oncology, University of Bern, 3010 Bern, Switzerland; 5 Charité—Universitätsmedizin Berlin, Corporate Member of Freie Universität Berlin and Humboldt-Universität zu Berlin, Department of Radiation Oncology, 13353 Berlin, Germany; felix.ehret@charite.de; 6 European Cyberknife Center, 81377 Munich, Germany; christoph.fuerweger@cyber-knife.net (C.F.); alexander.muacevic@cyber-knife.net (A.M.); 7 Department of Stereotaxy and Functional Neurosurgery, University of Cologne, Faculty of Medicine and University Hospital Cologne, 50937 Cologne, Germany; 8 Faculty of Medicine, University of Zurich, 8006 Zurich, Switzerland

**Keywords:** artificial intelligence, deep learning, machine learning, vestibular, schwannoma, neuro-oncology

## Abstract

**Introduction**: Many proposed algorithms for tumor detection rely on 2.5/3D convolutional neural networks (CNNs) and the input of segmentations for training. The purpose of this study is therefore to assess the performance of tumor detection on single MRI slices containing vestibular schwannomas (VS) as a computationally inexpensive alternative that does not require the creation of segmentations. **Methods**: A total of 2992 T1-weighted contrast-enhanced axial slices containing VS from the MRIs of 633 patients were labeled according to tumor location, of which 2538 slices from 539 patients were used for training a CNN (ResNet-34) to classify them according to the side of the tumor as a surrogate for detection and 454 slices from 94 patients were used for internal validation. The model was then externally validated on contrast-enhanced and non-contrast-enhanced slices from a different institution. Categorical accuracy was noted, and the results of the predictions for the validation set are provided with confusion matrices. **Results**: The model achieved an accuracy of 0.928 (95% CI: 0.869–0.987) on contrast-enhanced slices and 0.795 (95% CI: 0.702–0.888) on non-contrast-enhanced slices from the external validation cohorts. The implementation of Gradient-weighted Class Activation Mapping (Grad-CAM) revealed that the focus of the model was not limited to the contrast-enhancing tumor but to a larger area of the cerebellum and the cerebellopontine angle. **Conclusions**: Single-slice predictions might constitute a computationally inexpensive alternative to training 2.5/3D-CNNs for certain detection tasks in medical imaging even without the use of segmentations. Head-to-head comparisons between 2D and more sophisticated architectures could help to determine the difference in accuracy, especially for more difficult tasks.

## 1. Introduction

Even though the application of neural networks (NNs) for brain tumor detection on magnetic resonance imaging (MRI) could have the potential to improve patient safety when it serves as an automated second opinion or to increase the efficiency of physicians by taking over parts of the radiology workflow, there is still no established, or commercially available, solution that is able to detect and classify different brain tumor entities.

Many proposed detection as well as classification algorithms rely on 3D convolutional neural networks (CNNs) or 2.5D CNNs that include all or parts of the spatial information that is present in an MRI [1,2,3]. Indeed, a variety of publications report good performance of different 3D architectures, often for segmentation and subsequent feature extraction, which is used for classification tasks on the publically available Multimodal Brain Tumor Segmentation Challenge (BraTS) dataset [4,5,6]. 

While this has the advantage of improved performance due to the interslice context that a model can use for its predictions, it increases the computational power that is required, in particular for training, due to the higher number of weights that need to be stored and updated simultaneously in the graphical processing unit (GPU) memory [7]. 

Furthermore, many data augmentation techniques that are well established in the 2D space are less straightforward when the input data have three dimensions, which also applies to spatial normalization, especially when working with data from different institutions [8,9]. 

In addition, many algorithms require the input of segmentations, which supports the model regarding the area of the image that it needs to focus on but also increases the effort that is needed to label the training data since the creation of contours or bounding boxes tends to be more time-consuming than selecting the slices that contain tumor [2,10,11].

The purpose of this study was therefore to assess the feasibility of brain tumor detection on single slices using advances in transfer learning and data augmentation techniques but without the support of interslice context or segmentations. To keep the amount of required training data low, we chose a surrogate approach with limited scope as a first step, assuming that if the model were to fail on this task, more complex applications would fail as well. 

Since vestibular schwannomas (VS) can vary in shape and size but occur consistently in the cerebellopontine angle (CPA), they can be classified according to the side from which they arise (right vs. left) and therefore constitute an ideal tumor entity for the aforementioned approach before assessing it in on real detection tasks or other entities that can occur in different locations and are therefore likely to require more training data [12].

Herein we evaluate the performance of a 2D CNN that can be trained on small GPUs in a short time without the use of segmentations for classifying the laterality of vs. and use advances in explainable artificial intelligence to illustrate what parts of the image the model is using as a basis for its decision. 

## 2. Methods

In this retrospective study, 2992 patients containing VS were created from the MRIs of 633 patients provided by the European Cyberknife Center in Munich (Germany) and labeled according to the tumor location (right: 1415 images from 301 patients vs. left: 1577 images from 332 patients). 

A ResNet-34 pretrained on ImageNet was retrained as part of a transfer learning approach to classify the slices according to the tumor location [13]. The architecture was chosen for its demonstrated ability in image classification as well as due to the fact that a variety of ResNet architectures with different complexities were all pretrained on ImageNet are available to be imported in several popular deep learning libraries [14]. 

A total of 2538 slices from 539 patients were used as training data (right: 1231 slices from 261 patients; left: 1307 slices from 278 patients), and 454 slices from 94 patients were used for internal validation (right: 184 slices from 40 patients; left: 270 slices from 54 patients). To prevent data leakage, the slices of the training and the internal validation cohort were created from different patients. 

In a subsequent step, we conducted an external validation using 74 T1-weighted contrast-enhanced axial slices from 74 patients provided by the cantonal hospital St. Gallen (Switzerland) to assess the performance of the network and the possible presence of overfitting.

We implemented Grad-CAM to identify regions that the predictions of the network were based on [15].

To assess if the network was able to generalize to slices without contrast enhancement, we conducted a second external validation with 73 T1-weighted axial slices without contrast enhancement from 73 patients provided by the same institution. Since the two types of slices (with and without contrast enhancement) were not available for all patients, the total number of patients analyzed in the external validation cohorts of this study is actually 82 (65 patients were used to create contrast-enhanced as well as non-contrast-enhanced slices, 9 patients were only used to create contrast-enhanced slices, and 8 patients were only used to create non-contrast-enhanced slices).

Labeling of the slices used as training and internal validation data was done by a radiation oncology resident as well as a medical student from MRIs of patients who were diagnosed with vestibular schwannoma by a board-certified radiologist and referred for stereotactic radiotherapy by an interdisciplinary tumor board. The external validation data, both contrast-enhanced and non-contrast-enhanced, were sampled consecutively from the database of the radiology department of the cantonal hospital St. Gallen and either labeled or reviewed by two board-certified radiologists. Only tumors that had not received any kind of pretreatment were allowed in the external validation cohort so that the network could not use treatment-associated changes, such as postoperative scarring, for its predictions. However, patients with a history of radiotherapy (18 patients = 2.8%) or surgery to the tumor (112 patients = 17.7%) were allowed in the training as well as the internal validation cohort as long as the macroscopic tumor was visible on the slices. 

All slices were created randomly, sometimes containing only very small amounts of tumor while others contained the maximum diameter.

Programming was done using python (version 3.6) and the fastai (version 2) as well as PyTorch (version 1.7) libraries. All code that was used for training and validating the networks is provided as a supplemental file [16,17].

Data augmentation was performed with the fastai RandomResizedCrop (minimum scale = 0.9) and aug_transforms (max_lighting = 0.1, max_rotate = 15.0, do_flip = False) functions, with the latter providing rotation, zoom, and changes to brightness as well as contrast. All images were resized to 224 × 224 pixels prior to inputting them to the ResNet as a compromise between providing sufficient detail for the network and ensuring fast computations.

Training was performed using the fastai fine_tune method for a total of 20 epochs with a variable learning rate and training for the first 5 epochs while frozen while monitoring the training and validation loss to ensure that the training was neither ended prematurely nor done too long to avoid overfitting. Flattened cross-entropy loss was used as the loss function and Adam as the optimizer [18].

All imaging data were handled in accordance with institutional policies, and approval by an institutional review board was obtained for the training as well as the internal validation cohort from the Ludwig Maximilian University of Munich (project 20-437, 22 June 2020) for a project on outcome modeling after radiosurgery of which this study is a subproject. Ethical approval for the images in the validation cohort was waived by the Ethics Committee of Eastern Switzerland (EKOS 21/041, 9 March 2021) due to the fact that using single MRI slices constitutes sufficient anonymization. 

Written informed consent for the analysis of anonymized clinical and imaging data was obtained from all patients.

Confidence intervals of the performance on the test set were computed as CI = 1.96 × sqrt((accuracy × (1 − accuracy))/n), where n is the size of the respective test set. 

**Figure 1:**
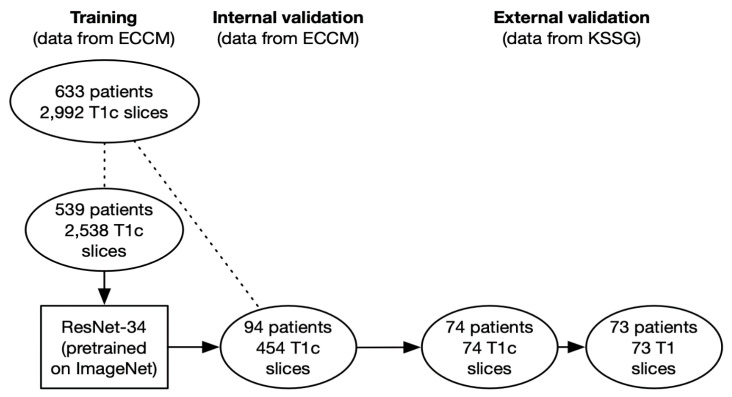
Training; internal and external validation workflow. ECCM = European Cyberknife Center in Munich. KSSG = Kantonsspital St. Gallen. Patients or slices connected by dotted lines belong to the same dataset.

The workflow is depicted in Figure 1.

**Figure 2:**
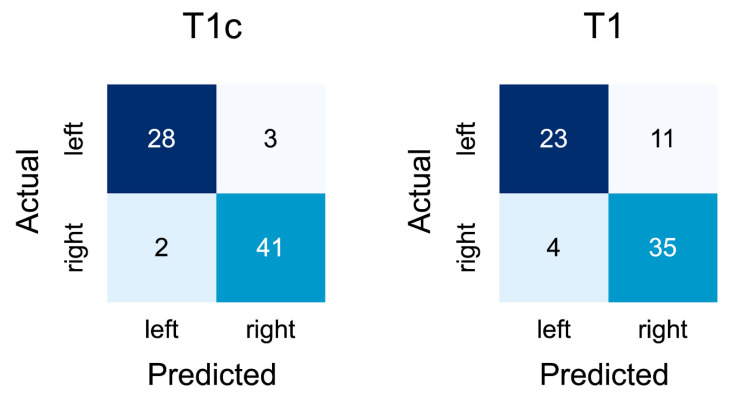
Confusion matrices for the performance of the network during external validation on axial T1-weighted slices with (left) and without (right) contrast-enhancement.

Training and validation losses for the network during the training process are depicted in Figure 2.

## 3. Results

The ResNet-34 achieved an accuracy of 0.974 (95% CI: 0.960–0.988) on the internal validation cohort during retraining. 

**Figure 3:**
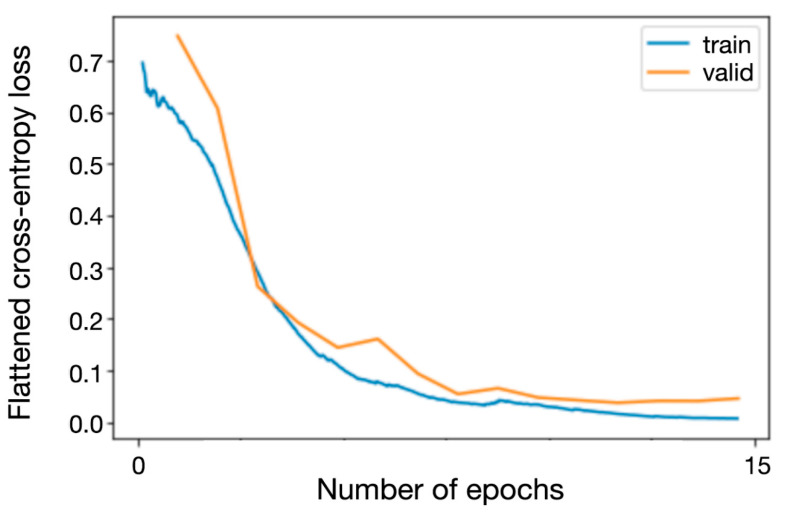
Training (blue) and internal validation (yellow) loss by training epoch for the 15 unfrozen epochs.

On the contrast-enhanced slices of the external validation cohort, the network achieved an accuracy of 0.928 (95% CI: 0.869–0.987). A confusion matrix for the results is depicted in Figure 3.

**Figure 4:**
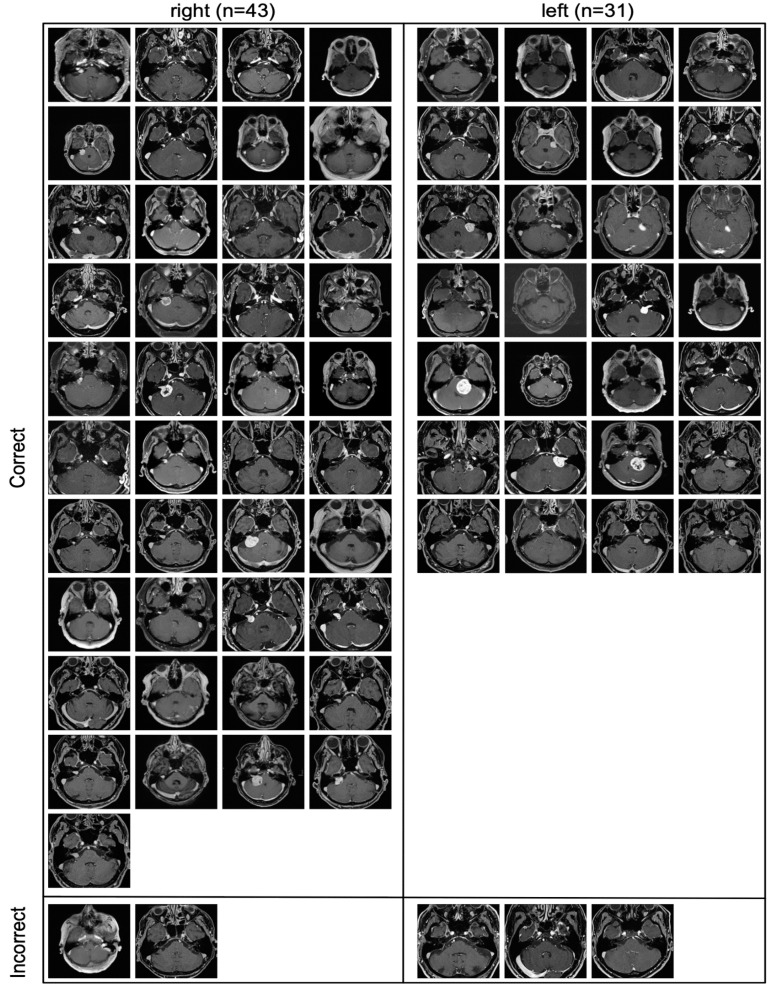
All images of the contrast-enhanced external validation cohort. All incorrectly classified slices (bottom) contained only a very small amount of tumor.

All the slices that were part of the contrast-enhanced external validation cohort are depicted in Figure 4, including whether they were classified correctly or incorrectly.

All incorrectly classified slices contained only a very small amount of tumor.

**Figure 5:**
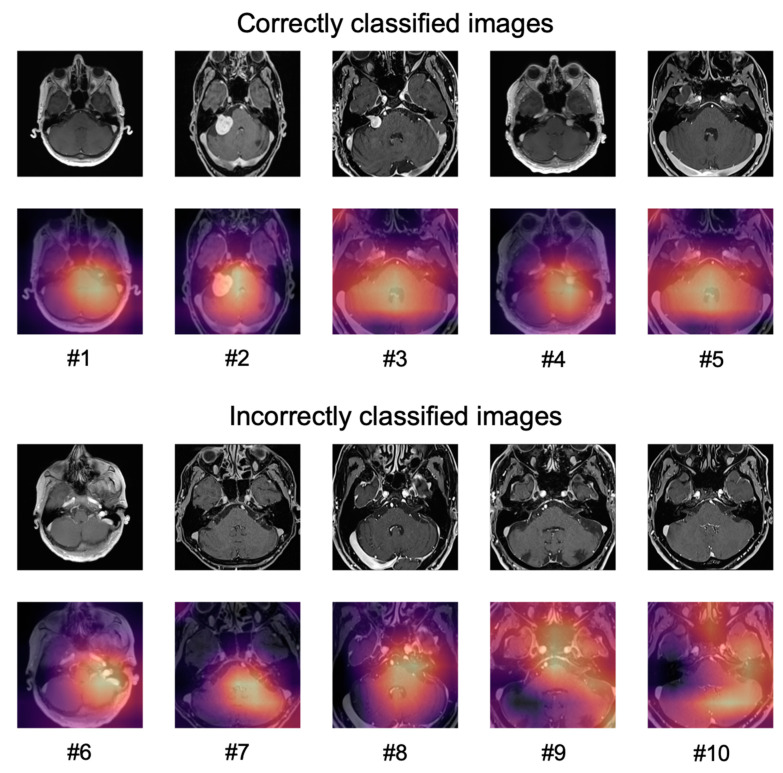
Correctly (top) and incorrectly (bottom) classified sample images from the contrast-enhanced validation cohort with and without Grad-CAM. For correctly classified images, the network did not seem to focus only on the tumor but on a larger part of the cerebellum and the cerebellopontine angle (#1–#5). For the incorrectly classified images, the network seemed to focus either on areas of the brain that contained blood vessels (patients #6–#8) or focused on larger areas of the image (patients #9, #10).

The application of Grad-CAM to visualize the areas of an image that the predictions of the network were based on, is shown for ten sample images in Figure 5.

For correctly classified images, the network did not seem to focus only on the tumor but on a larger part of the cerebellum and the cerebellopontine angle (#1–#5).

For the incorrectly classified images, the network seemed to focus either on areas of the brain that contained blood vessels (patients #6–#8) or focused on larger areas of the image (patients #9, #10).

Since the focus of the network was not limited to the contrast-enhancing tumor, we hypothesized that the network might be able to recognize the shapes that indicate the presence of a tumor and not be entirely reliant on the contrast enhancement. We therefore conducted another external validation on T1-weighted axial slices without contrast enhancement where the network, although being trained only on images with contrast enhancement, achieved an accuracy of 0.795 (95% CI: 0.702–0.888).

**Figure 6:**
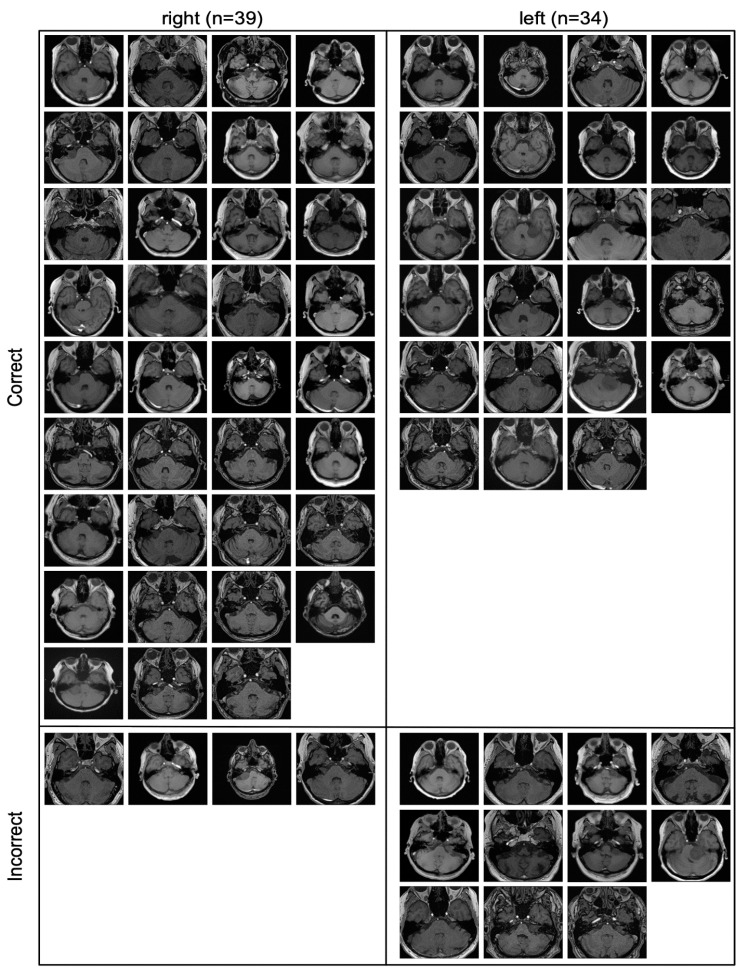
All images of the non-contrast-enhanced external validation cohort. Incorrectly classified slices (bottom) contain both schemes.

All the slices that were part of the non-contrast-enhanced external validation cohort are depicted in Figure 6, including whether they were classified correctly or incorrectly.

Notably, the incorrectly classified images were more heterogeneous in the non-contrast-enhanced cohort with both smaller and larger tumors being present.

## 4. Discussion

In this study, an NN was able to classify contrast-enhanced slices of VS according to the side of the tumor with a high accuracy that could be sustained when deploying the network to slices from a different institution with only a slightly reduced accuracy.

Data to compare the achieved accuracy to are limited. Searching PubMed on 7 March 2021 using a broad query (“((vestibular schwannoma[Title]) OR (vestibular schwannomas[Title])) AND ((deep[Title]) OR (network[Title]) OR (networks[Title]) OR (artificial intelligence[Title]))”) yielded only nine results, all of which were published between 2014 and 2021. Four of those results were unrelated to radiology [19,20,21,22]. One publication used an NN to predict vs. recurrence following surgery from clinical parameters in tabular format [23]. The remaining four publications used NNs to segment VS for either radiotherapy planning or response assessment [24,25,26,27].

The high accuracy of the validation data can likely be attributed to a combination of both the heterogenous training data that, while being provided from one institution, contained studies acquired on a variety of different scanners and the variety of data augmentation techniques that were used. The fact that the network failed only on slices that showed a very small amount of tumor can be considered encouraging as well. Most modern MRIs acquire fairly thin slices < 2 mm for T1-weighted sequences so that there will almost always be slices containing larger parts of the tumor that can be correctly classified with higher confidence except for possibly the smallest cases of VS.

In addition, the confusion matrices indicate that the network did not seem biased towards one side for T1-weighted contrast-enhanced slices but was slightly more inclined to incorrectly predict left-sided tumors as being right-sided than vice versa, though it is unknown whether this would also be the case in a larger external validation set (Figure 3).

The correctly and incorrectly classified slices in Figure 4 indicate that the model mainly failed when confronted with slices where very little tumor is present, which can be the case with very small tumors or on the edge of a tumor.

However, one has to acknowledge that classifying slices according to the tumor location is only a surrogate for classifying slices according to whether or not they contain tumor, which is the real clinical use case. It would have been more optimal to train and test the network on slices containing VS in contrast to healthy slices from the cerebellopontine angle, but this would have required access to an equally large as well as heterogeneous dataset of patients without VS or another pathology. More information on whether classifying laterality is a viable surrogate for detection in case of VS could be obtained by splitting each image used for training and validation into a “healthy” and a “VS” hemisphere and trying to classify the newly created images accordingly, which will be a follow-up project to this study.

The main question for future studies will be whether predictions on 2D slices will be able to achieve accuracies comparable to 3D-CNNs when deployed on whole MRIs for detection tasks. 

While 3D-CNNs are likely to always remain somewhat better due to the additional information provided by the interslice context, using predictions on 2D slices might improve to a point where a slight reduction in accuracy is outweighed by the reduction in computational power that is required. While the inference time of 2D- and 3D-CNNs might not differ all that much, simultaneously updating all the weights of a 3D-CNN during training requires a more powerful GPU, which may be an obstacle especially when trying to conduct a series of experiments.

Labeling data by simply selecting the appropriate slices instead of creating segmentations could shorten the development process of new models as well, though techniques such as semi-automatic segmentations are also contributing to decreasing the time that is required for the latter [28].

Labeling without supporting segmentations or bounding boxes is of course more likely to be successful for simpler tasks such as the detection of VS as compared, for example, to the detection of brain metastases that can vary significantly with regard to location and appearance.

In addition, one might also implement a very basic as well as computationally inexpensive way to benefit from interslice context for an architecture using 2D predictions, e.g., by using similar predictions on adjacent slices to obtain the prediction for the whole MRI.

However, there are studies indicating that for some tasks, 3D CNNs, in particular ensembles of different 3D CNNs, may sustain superior performance compared to 2D CNNs [29].

Another interesting question is whether the performance of the network would have been better by using only slices of previously untreated VS for training. While this would have reduced the number of training data available to the model, the images themselves would have been more similar to the images that the model was then tested on. 

The fact that the performance of the model could partly be sustained on non-contrast-enhanced slices from a different institution is another interesting finding of this study and could serve as a foundation for other studies to explore to what extent networks trained on slices from one acquisition sequence are able to generalize to slices from another one as well as how well the sequences perform when trying to classify the laterality of VS. However, it cannot be excluded that a weak contrast enhancement on some slices from the training data might have helped the model’s performance on the non-contrast-enhanced validation cohort.

The use of Grad-CAM in this study provided important information and led us to evaluate the performance of the model on non-contrast-enhanced slices. Explainable AI has seen increased use in machine learning in general as well as machine learning in medicine in particular and has been shown to benefit the development of models in various ways [30]. Furthermore, understanding the decisions of a model is important to enable the adoption of a model by clinicians as they are less likely to use something that is deemed a “black box” [31].

Limitations of this study include the aforementioned use of classifying location as a surrogate for detection and the fact that no data on the performance 3D-CNNs for the same task are available. In addition, no quantitative assessment of the Grad-CAM images has been performed. Strengths include the independent validation cohorts with a significant number of patients as well as the heterogeneous training data. 

## 5. Conclusions

This study shows that classifying the laterality of vestibular schwannomas on single MRI slices without the use of segmentations is feasible and achieved an accuracy of 0.928 with the data and training procedure that was described. Single slice predictions might constitute a computationally inexpensive alternative to training 2.5/3D-CNNs for certain detection tasks in medical imaging even without the use of segmentations, possibly enabling more efficient data labeling and model training. Head-to-head comparisons between 2D and more sophisticated architectures could help to determine the difference in accuracy, especially for more difficult tasks. In addition, the validity of classifying laterality as a surrogate for detection needs to be investigated. 

## Data Availability

The data presented in this study are available from the corresponding author on reasonable request.

## Supplementary Materials

The following are available online at https://www.mdpi.com/article/10.3390/diagnostics11091676/s1.

## Code Availability

All code that was used for training and validating the networks is provided as a supplemental file.
