# Supplementary material for: Convolutional Neural Networks for Classifying Laterality of Vestibular Schwannomas on Single MRI Slices—A Feasibility Study
Source: Diagnostics (Basel). 2021 Sep 14;11(9):1676. doi: 10.3390/diagnostics11091676 (PMC8465488; doi:10.3390/diagnostics11091676)
Supplement: Supplementary file 1 [file diagnostics-11-01676-s001.zip › diagnostics-1330899-supplementary.pdf]

```python
#!/usr/bin/env python
# coding: utf-8

# <h1>Table of Contents<span class="tocSkip"></span></h1>
# <div class="toc"><ul class="toc-item"><li><span><a href="#Setup" data-toc-modified-id="Setup-1"><span class="toc-item-
num">1  </span>Setup</a></span></li><li><span><a href="#Read-data" data-toc-modified-id="Read-data-2">
<span class="toc-item-num">2  </span>Read data</a></span><ul class="toc-item"><li><span><a href="#Label-
data" data-toc-modified-id="Label-data-2.1"><span class="toc-item-num">2.1  </span>Label data</a></span></li>
<li><span><a href="#Check-datablock" data-toc-modified-id="Check-datablock-2.2"><span class="toc-item-
num">2.2  </span>Check datablock</a></span></li></ul></li><li><span><a href="#Train-Models" data-toc-
modified-id="Train-Models-3"><span class="toc-item-num">3  </span>Train Models</a></span><ul class="toc-
item"><li><span><a href="#ResNet-34" data-toc-modified-id="ResNet-34-3.1"><span class="toc-item-num">3.1  
</span>ResNet-34</a></span></li></ul></li><li><span><a href="#Export-Models" data-toc-modified-id="Export-Models-4">
<span class="toc-item-num">4  </span>Export Models</a></span></li><li><span><a href="#Interpret-Models" data-
toc-modified-id="Interpret-Models-5"><span class="toc-item-num">5  </span>Interpret Models</a></span><ul
class="toc-item"><li><span><a href="#ResNet-34" data-toc-modified-id="ResNet-34-5.1"><span class="toc-item-
num">5.1  </span>ResNet-34</a></span></li></ul></li><li><span><a href="#External-validation" data-toc-modified-
id="External-validation-6"><span class="toc-item-num">6  </span>External validation</a></span><ul class="toc-
item"><li><span><a href="#ResNet-34" data-toc-modified-id="ResNet-34-6.1"><span class="toc-item-num">6.1  
</span>ResNet-34</a></span></li></ul></li><li><span><a href="#T1-without-contrast" data-toc-modified-id="T1-without-
contrast-7"><span class="toc-item-num">7  </span>T1 without contrast</a></span><ul class="toc-item"><li>
<span><a href="#ResNet-34" data-toc-modified-id="ResNet-34-7.1"><span class="toc-item-num">7.1  
</span>ResNet-34</a></span></li></ul></li></ul></div>

# # Setup

# In[1]:


from fastai.vision.all import*
from fastai.callback.fp16 import*


# # Read data

# In[2]:


path = untar_data("VS_LvsR")
path.ls()


# ## Label data

# In[3]:


vsSlices = DataBlock(blocks = (ImageBlock, CategoryBlock),
                   get_items=get_image_files,
                   splitter=GrandparentSplitter(train_name='train', valid_name='valid'),
                   get_y=parent_label,
                    item_tfms=RandomResizedCrop(224, min_scale=0.9),
                    batch_tfms=aug_transforms(max_lighting=0.1, max_rotate=15.0 ,do_flip=False))
dls_vsSlices = vsSlices.dataloaders(path)


# ## Check datablock

# In[4]:


dls_vsSlices.show_batch(nrows=2, ncols=3)


# # Train Models

# ## ResNet-34
```

```python
# In[54]:


learn_vsSlices34 = cnn_learner(dls_vsSlices, resnet34, metrics=accuracy).to_fp16()
learn_vsSlices34.fine_tune(15, freeze_epochs=5)


# # Export Models

# In[20]:


learn_vsSlices34.export("vsSlices34.pkl")


# # Interpret Models

# ## ResNet-34

# In[24]:


interp34 = ClassificationInterpretation.from_learner(learn_vsSlices34)
interp34.plot_top_losses(k = 10,nrows = 2,largest=True)


# In[26]:


interp34.plot_confusion_matrix(figsize=(4,4), dpi=60)


# # External validation

# In[29]:


validPath = untar_data("VS_AX_T1c_valid")
validFiles = get_image_files(validPath)


# In[30]:


validFiles


# In[31]:


testdl = dls_vsSlices.test_dl(validFiles, with_labels=True)


# ## ResNet-34

# In[143]:


learn_vsSlices34.validate(dl=testdl)


# In[144]:


interp_VS34_valid = ClassificationInterpretation.from_learner(learn_vsSlices34, dl=testdl)
interp_VS34_valid.plot_confusion_matrix(figsize=(4,4), dpi=120)
```

```
# In[142]:


interp_VS34_valid.plot_top_losses(k = 10,nrows = 2,largest=True)


# # T1 without contrast

# In[41]:


validNonContrastPath = untar_data("VS_AX_T1_valid")
validNonContrastFiles = get_image_files(validNonContrastPath)


# In[42]:


validNonContrastFiles


# In[44]:


testNonContrastdl = dls_vsSlices.test_dl(validNonContrastFiles, with_labels=True)


# ## ResNet-34

# In[145]:


learn_vsSlices34.validate(dl=testNonContrastdl)


# In[146]:


interp_VS34_NonContrast = ClassificationInterpretation.from_learner(learn_vsSlices34, dl=testNonContrastdl)
interp_VS34_NonContrast.plot_confusion_matrix(figsize=(4,4), dpi=120)


# In[64]:


learn_vsSlices34.recorder.plot_loss(skip_start=0)


# In[ ]:
```
